# Calidad de vida y estado funcional al egreso hospitalario de pacientes con COVID-19 en Colombia

**DOI:** 10.15446/rsap.V25n3.107343

**Published:** 2023-05-01

**Authors:** Emily C. Mejía-Fique, Jorge E. Gerónimo-Malaver, Jennyfer Y. Ospina-Olarte, Olga J. Gómez-Ramírez, Hernando G. Gaitán-Duarte

**Affiliations:** 1 EM: Enf. Investigadora. Facultad de Enfermería. Universidad Nacional de Colombia. Bogotá, Colombia. ecmejiaf@unal.edu.co Universidad Nacional de Colombia Facultad de Enfermería Universidad Nacional de Colombia Bogotá Colombia ecmejiaf@unal.edu.co; 2 JG: Enf. Investigador. Facultad de Enfermería. Universidad Nacional de Colombia. Bogotá, Colombia. jegeronimom@unal.edu.co Universidad Nacional de Colombia Facultad de Enfermería Universidad Nacional de Colombia Bogotá Colombia jegeronimom@unal.edu.co; 3 JO: Enf. Investigadora. Facultad de Enfermería. Universidad Nacional de Colombia. Bogotá, Colombia. jyospinao@unal.edu.co Universidad Nacional de Colombia Facultad de Enfermería Universidad Nacional de Colombia Bogotá Colombia jyospinao@unal.edu.co; 4 OG: Enf. M.Sc. Enfermería. Ph. D. Enfermería. Facultad de Enfermería. Universidad Nacional de Colombia. Bogotá, Colombia. ojgomezr@unal.edu.co Universidad Nacional de Colombia Facultad de Enfermería Universidad Nacional de Colombia Bogotá Colombia ojgomezr@unal.edu.co; 5 HG: MD. M. Sc. Epidemiología Clínica. Facultad de Medicina. Universidad Nacional de Colombia. Hospital Universitario Nacional de Colombia. Bogotá, Colombia. hggaitand@unal.edu.co Universidad Nacional de Colombia Epidemiología Clínica Facultad de Medicina Universidad Nacional de Colombia Bogotá Colombia hggaitand@unal.edu.co

**Keywords:** COVID-19, síndrome post-agudo de COVID-19, calidad de vida relacionada con la salud, estado funcional *(fuente: DeCS, BIREME)*, COVID-19, post-acute COVID-19 syndrome, health-related quality of life, functional status *(source: MeSH, NLM)*

## Abstract

**Objetivo:**

Determinar la percepción de calidad de vida y el estado funcional tras el egreso hospitalario de personas colombianas con COVID-19.

**Materiales y Métodos:**

Se realizó un estudio cuantitativo transversal descriptivo en el que se aplicó el cuestionario SF-36 y la escala del estado funcional post-COVID-19 a personas colombianas en el periodo post-egreso hospitalario para evaluar la calidad de vida y la funcionalidad después de la COVID-19.

**Resultados:**

Se contó con 151 participantes, con edad media de 56,43 años, el 68% eran hombres. El 79% estuvo en unidad de cuidado intensivo, el promedio de estancia hospitalaria fue de 28,46 días, el 22% tenía el hábito de fumar y el 64% tenían comorbilidades. Se evidenció una disminución en la percepción de calidad de vida relacionada con la salud, principalmente en las dimensiones de vitalidad (0,47), salud general (0,56) y rol físico (0,56). Se evidenció presencia de limitaciones funcionales que iban desde mínimas a severas. Los síntomas persistentes más frecuentes fueron fatiga, disnea y dolor.

**Conclusiones:**

La COVID-19 tiene un impacto importante en la percepción de la calidad de vida y la funcionalidad de pacientes sobrevivientes, incluso más allá de seis meses tras el alta hospitalaria.

## Abstract (en)

**Objective:**

To determine the perception of quality of life and functional status after hospital discharge in Colombian patients with COVID-19.

**Materials and Methods:**

A quantitative cross-sectional descriptive study was carried out in which the SF-36 questionnaire and the post-COVID-19 functional status scale were applied to Colombians in the post-hospital discharge period to assess quality of life and functionality after COVID-19.

**Results:**

There were 151 participants, with a mean age of 56.43 years, 68% of whom were men. 79% were in the Care Unit, the average hospital stay was 28.46 days, 22% were smokers, and 64% had comorbidities. There was evidence of a decrease in the perception of health-related quality of life, mainly in the dimensions of vitality (0.47), general health (0.56), and physical role (0.56). There was evidence of functional limitations ranging from minimal to severe. The most frequent persistent symptoms were fatigue, dyspnea, and pain.

**Conclusions:**

COVID-19 impacts the perception of quality of life and functionality of surviving patients, even beyond six months after hospital discharge.

La COVID-19 es una enfermedad de origen viral que puede tener implicaciones en diferentes órganos y sistemas del cuerpo humano, aunque afecta principalmente al aparato respiratorio 1. Su sintomatologia es heterogénea ya que varía de acuerdo a las características del huésped, el virus y el entorno, por lo que puede tener diversas formas de presentación con manifestaciones leves, moderadas, graves o críticas 1.

La propagación del virus SARS-CoV-2, responsable de la COVID-19, ha convocado a nivel mundial los esfuerzos de la comunidad científica para la generación de conocimiento que ayude a mitigar el impacto de la emergencia sanitaria. En la actualidad, el seguimiento a los recuperados de la COVID-19 y la atención e identificación de las consecuencias derivadas de la enfermedad son el nuevo reto 2. Sobre este tema, las investigaciones llevadas a cabo con los pacientes post-COVID-19 aún son limitadas en número y se han desarrollado en contextos significativamente diferentes a los de nuestro país. No obstante, algunos de estos estudios ya evidencian consecuencias negativas de la enfermedad en el bienestar general de los pacientes tras el alta hospitalaria.

Nuestro estudio indagó sobre la calidad de vida relacionada con la salud (CVRS) y el estado funcional de los pacientes post-COVID-19. Estas variables también se han investigado en otros estudios 2-17 que se han desarrollado y publicado recientemente en distintos países, incluyendo Países Bajos, Alemania, España, Austria, Italia, Francia, Bélgica, Noruega, Dinamarca, Portugal, Suiza, Canadá, Estados Unidos de América, Brasil y China. Los resultados de estas investigaciones han descrito la afectación significativa que genera la COVID-19 sobre la calidad de vida evaluada en periodos posteriores al alta hospitalaria, principalmente debido a la persistencia de síntomas desagradables asociados a la enfermedad como la fatiga, la disnea, el dolor, la alteración del patrón del sueño, la pérdida de la memoria, la depresión y la ansiedad, así como las alteraciones sobre el funcionamiento físico y social.

De la misma manera, los hallazgos referentes a la evaluación funcional post-aguda de los pacientes con COVID-19 han demostrado el importante deterioro sobre el estado funcional derivado de la enfermedad al identificar una gran proporción de pacientes con limitaciones a los seis meses del alta hospitalaria 9. Adicionalmente, se conoce que la calidad de vida está estrechamente relacionada con la percepción del estado funcional de los pacientes, por lo cual la evaluación de ambas variables es fundamental, sobre todo ante problemas de salud significativos 18.

El objetivo de esta investigación fue determinar la percepción de la calidad de vida relacionada con la salud y el nivel de funcionalidad tras el egreso hospitalario de los pacientes diagnosticados y tratados por COVID-19 en el Hospital Universitario Nacional de Colombia (HUN), ubicado en la ciudad de Bogotá, Colombia.

## MÉTODOS

Se realizó un estudio con enfoque y alcance cuantitativo-descriptivo, de diseño no experimental y corte transversal, para describir la calidad de vida relacionada con la salud y la funcionalidad de pacientes colombianos en un periodo posterior a seis meses de la etapa aguda de la enfermedad por COVID-19. Para la selección de los participantes se contó con una base de datos de egreso hospitalario facilitada por el HUN que contaba con 284 registros entre abril de 2020 y agosto de 2021. Los criterios de inclusión de participantes fueron: personas mayores de 18 años que requirieron cuidado intrahospitalario en el HUN para el manejo de COVID-19 y que al momento del diligenciamiento ya hubieran recibido egreso hospitalario; personas con capacidad de responder el cuestionario y deseo de participar en el estudio. Se excluyeron las personas en condición de discapacidad que no pudieran dar respuesta a las preguntas. Para la aplicación de los instrumentos se siguió un protocolo de recolección de datos y se contactó a los participantes mediante llamada o videoconferencia Los instrumentos aplicados fueron: ficha de caracterización sociodemográfica construida por los investigadores, el cuestionario 36-Item Short Form Survey (SF-36) -adaptado lingüística y culturalmente para Colombia- y la Escala de Estado Funcional Post COVID-19 (PCFS), también validada para su uso en el país.

Se llevó a cabo una prueba piloto con 15 participantes, correspondientes al 10% de la población del estudio principal, que permitió ajustar metodológicamente el estudio y contribuyó a la disminución de sesgos y errores en la recolección de datos. Los datos fueron codificados para garantizar la confidencialidad y la privacidad de los participantes y fueron analizados y descritos mediante el uso del software estadístico R.

El estudio fue avalado por el Comité de Ética de la Facultad de Medicina de la Universidad Nacional de Colombia y el comité del HUN, mediante las actas 009-064 y CEI-HUN-ACTA-2021-07, respectivamente. Adicionalmente, se contó con aprobación para el uso de los instrumentos por parte de los autores.

### Cuestionario 36-Item Short Form Survey (SF-36)

El cuestionario 36-Item Short Form Survey (SF-36) es un instrumento genérico que mide la CVRS. Fue desarrollado originalmente en Estados Unidos en el marco del Estudio de los Resultados Médicos (Medical Outcomes Study, por su nombre en inglés) por Ware y Sherbourne en 1992 19. Cuenta con un total de 36 ítems distribuidos en 8 dimensiones: funcionamiento físico (FF), desempeño físico (DF), dolor corporal (DC), desempeño emocional (DE), salud mental (SM), vitalidad (V), salud general (SG) y funcionamiento social (FS). El cuestionario se destaca por su brevedad y fácil comprensión, otorga un resultado entre 0 puntos, que representa el peor estado de salud percibida, y 100 puntos, que corresponde al mejor estado de salud percibida. Adicionalmente, tiene en cuenta la evaluación del cambio de la salud en el tiempo, un indicador que no otorga puntuación, pero es relevante para la evaluación de la CVRS 20. Es una de las escalas genéricas más utilizadas en estudios descriptivos y es reconocida para evaluar el impacto de cualquier enfermedad, sus terapéuticas y la atención en salud sobre la vida de los pacientes 21-22.

En Colombia solo un estudio da cuenta de las propiedades psicométricas del cuestionario aplicado en el territorio nacional, en este, los autores reportan que *en* todas las dimensiones (FF, DF, DC, DE, SM, V, SG y FS) el coeficiente alfa de Cronbach superó el estándar de 0,70 y resaltaron su validez de apariencia, contenido, criterio y constructo 23. Estos resultado s informan que el cuestionario brinda una adecuada confiabilidad de constructo y muestran una homogeneidad respecto a la correlación de los 36 ítems para evaluar el mismo concepto, lo cual demuestra que es una escala fiable para su uso en la determinación de la CVRS 23.

### Escala de Estado Funcional Post COVID-19 (PCFS)

La Escala de Estado Funcional Post COVID-19 (PCFS, por sus siglas en inglés) es una escala ordinal desarrollada por Klok et al. en el marco del Post Covid-19 Functional Status Project 24. Es hasta el momento el único instrumento que evalúa el estado funcional resultante de esta patología 25 y ha demostrado utilidad para la identificación de las limitaciones funcionales derivadas de la COVID-19, siendo un instrumento valio o para complementar los resultados de otras evaluaciones más complejas como la de CVRS y la de sintomatología.

Dada la gran heterogeneidad en las formas de presentación de la COVID-19, el instrumento tiene en cuenta la gama completa de limitaciones funcionales posibles, incluyendo los cambios en estilo de vida, las limitaciones en la ejecución de actividades o tareas relacionadas con los distintos entornos en los que se desarrolla el sujeto, el hogar, el estudio o el trabajo y la sintomatología asociada 24.

La escala cuenta con un total de 17 ítems referidos a la situación del paciente en la última semana, a partir de los cuales se puede realizar una clasificación del grado de limitación funcional de la persona, donde 0= sin limitaciones funcionales; 1= limitaciones funcionales mínimas; 2= limitaciones funcionales ligeras; 3= limitaciones funcionales moderadas, 4= limitaciones funcionales severas y D = muerte. La PCFS cuenta con dos versiones, una para diligenciar a través de entrevista formal y otra de diligenciamiento autorreportado como versión para el paciente 24,26.

Hasta el momento, la escala cuenta con más de 10 traducciones a distintos idiomas y diferentes validaciones culturales para su uso alrededor del mundo ^(27^). En particular, para Colombia ya se encuentra validada para su uso clínico y en investigación por Benavides et al. 26.

## RESULTADOS

Se contó con una base de egreso hospitalario del HUN con el registro de 284 pacientes que fueron dados de alta entre abril de 2020 y agosto de 2021. De los pacientes registrados, 27 no cumplían con los criterios de inclusión, 35 habían fallecido, 2 tenían registro duplicado y 69 no fueron incluidos en d estudio por imposibilidad de contacto. En total se contó con 151 participantes a quienes se les aplicaron los cuestionarios.

**Figura 1:**
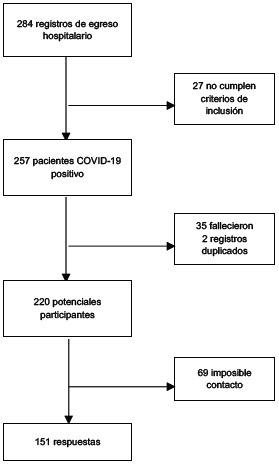
Diagrama de flujo de participantes del estudio

El 68% de los pacientes fueron hombres y el 32% mujeres, cuyas edades oscilaron entre los 27 y los 88 años, con una media de 56,43 años. Con respecto al estado civil, el 46% eran casados, el 25% vivían en unión libre y el 17% eran solteros, mientras que el porcentaje restante se encontraban divorciados o eran viudos.

De los 151 participantes, el 79% estuvieron hospitalizados en la unidad de cuidado intensivo (UCI), de los cuales la mayoría (72%) eran hombres. De los participantes restantes, el 17% estuvo en el servicio de hospitalización general y en su mayoría eran mujeres (56%). Finalmente, el 4% requirió atención en la unidad de cuidados intermedios (UCIN). De manera adicional, se evidenció que del total de participantes, 34 tenían el hábito de fumar, y de ellos el 88% requirió cuidado en UCI, lo que refleja un mayor nivel de complejidad condicionada por este factor. El promedio de días de estancia hospitalaria para el total de pacientes fue de 28,46 días, con un mínimo de 3 y un máximo de 174 días y una desviación estándar de 22,08. No se encontró relación significativa entre el sexo de los pacientes y el servicio requerido, ni entre el sexo y el número de días de estancia hospitalaria. Por el contrario, se evidenció una relación directa entre el hábito de fumar y los días de estancia hospitalaria.

En cuanto a comorbilidades, el 64% de los pacientes refirieron tener al menos una enfermedad diagnosticada, con mayor predominancia del grupo de enfermedades metabólicas, endocrinas o nutricionales con el 58%, donde resaltó la diabetes mellitus tipo II. Después de este se posicionó el grupo de enfermedades cardiovasculares con el 22%, donde tuvo mayor prevalencia la hipertensión arterial y con menor porcentaje enfermedades neurológicas, pulmonares y autoinmunes, entre otras.

En la etapa de rehabilitación tras el alta hospitalaria, 110 participantes requirieron algún tipo de intervención terapéutica, donde las más frecuentes fueron terapia respiratoria y física con el 59% y el 56%, respectivamente, seguido de otras terapias como la nutricional, la ocupacional, la psicológica y la fonoaudiológica. En el número de terapias recibidas por cada participante, el 22% recibió al menos una de las anteriores, el 25% recibió al menos dos terapias y el 11% recibió tres o cuatro terapias, siendo los de menor valor porcentual aquellos que requirieron cinco o seis terapias. Se encontró que a medida que aumentaron los días de estancia hospitalaria, aumentaba el número de terapias requeridas en la rehabilitación.

Los resultados del cuestionario SF-36 demuestran que la dimensión de vitalidad fue la más afectada (0,47), lo cual ocasionó que los participantes se sintieran cansados y exhaustos con frecuencia. Con respecto a la salud general y el rol físico (0,56, respectivamente), se encontró que los participantes identificaron problemas en el desarrollo de actividades de la vida diaria, incluyendo el trabajo, y evaluaron como regular la propia salud, con posibilidad de que esta empeorara. La función física (0,61) y la salud mental (0,65) evidencian que los participantes se han sentido un poco limitados para llevar a cabo actividades físicas como ducharse y han experimentado sentimientos de angustia y síntomas depresivos en el periodo posterior al egreso hospitalario. Algunos pacientes refirieron tener algún tipo de dolor que limita la ejecución de actividades cotidianas o laborales. Finalmente, se evidenció menor grado de afectación en la dimensión de rol emocional (0,77) y función social (0,8), donde los problemas emocionales o físicos en la etapa post COVID-19 no interfirieron en la capacidad de los participantes para participar en actividades sociales.

Sin embargo, de forma general, los resultados globales del cuestionario SF-36 demuestran que el valor promedio de calidad de vida percibida por los participantes fue de 65%, lo cual indica una buena calidad de vida percibida. No se observó relación estadísticamente representativa entre la edad de los participantes, el IMC y los días de estancia hospitalaria con el impacto en la calidad de vida percibida.

Por otro lado, los resultados de la Escala de Estado Funcional Post COVID-19 (PCFS) evidencian que del total de participantes, el 30% tuvieron limitaciones funcionales severas, el 15% tuvieron limitaciones funcionales moderadas, el 38% limitaciones funcionales ligeras, el 8% limitaciones funcionales mínimas y el 9% indicaron no tener limitaciones funcionales.

Se encontró que la fatiga fue el síntoma limitante más frecuente en los sobrevivientes de la COVID-19, referido por 74 participantes, seguido de disnea, dolor en miembros inferiores, dolor articular, limitación en la movilidad, parestesia en miembros inferiores, dolor lumbar, vértigo, dolor torácico, déficit de atención, dolor de cabeza, síntomas depresivos, disminución de la agudeza visual, disminución de la capacidad de memoria, dolor generalizado, parestesia en miembros superiores y tos.

Con relación a los síntomas no limitantes, los participantes refirieron como el síntoma más frecuente la tos, seguido de caída del cabello, irritabilidad, insomnio, cansancio, disfonía, disminución de la capacidad de memoria, ansiedad, inflamación en miembros inferiores, miedo, nerviosismo, odinofagia, síntomas depresivos y sudoración.

Un análisis de correlación entre la calidad de vida percibida y el estado funcional post-COVID-19 demuestra una relación inversamente proporcional, la cual refleja que a mayor puntuación en la escala PCFS, es menor la puntuación SF-36, lo que se traduce en que a mayor nivel de limitación funcional, menor es la calidad de vida percibida.

**Figura 2:**
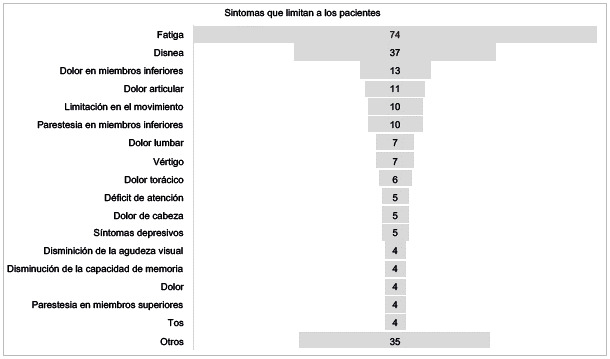
Síntomas post-COVID-19 que limitan a los participantes

**Figura 3:**
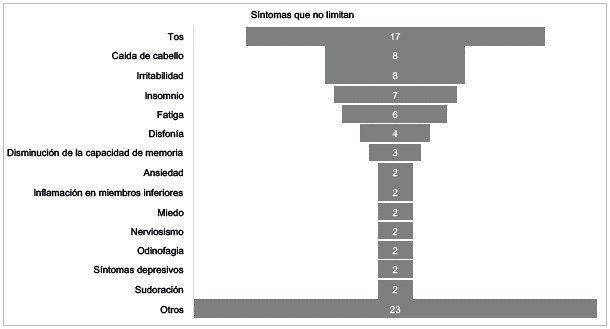
Síntomas post-COVID-19 que no limitan a los participantes

**Figura 4:**
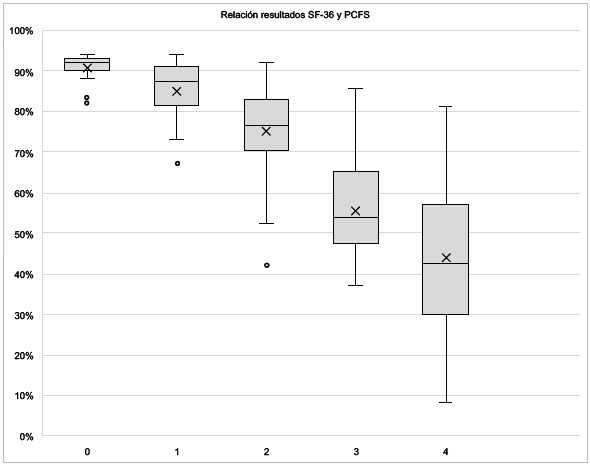
Relación Estado funcional post-COVID-19 - Calidad de vida percibida (instrumento SF-36)

Un análisis de correlación entre el valor de PCFS evidenció que a mayor número de días de estancia hospitalaria, mayor era la severidad de la limitación física en los participantes.

## DISCUSIÓN

Hasta el momento, este es el primer estudio reportado sobre la evaluación de la CVRS y el estado funcional de personas colombianas sobrevivientes de la COVID-19. Puntualmente en lo que respecta a la CVRS, esta investigación encontró que, de forma general, las personas encuestadas perciben una buena calidad de vida; sin embargo, se evidenciaron algunos deterioros en ciertos dominios evaluados, como el de la vitalidad. Esto es coherente con los resultados reportados por un estudio alemán a partir del cual se encontró que aun cuando muchos pacientes manifiestan malestar por la síntoma e etología asociada a la etapa post-COVID-19, en la mayoría de los casos esto no da lugar a repercusiones significativas sobre la calidad de vida general, pero sí a una afectación sobre algunas de las dimensiones evaluadas 28.

Por ejemplo, estudios elaborados en países europeos 16,29 que han utilizado los instrumentos EQ-5D-3L y EQ-5D-5L han demostrado que los sobrevivientes de COVID-19 generalmente manifiestan un empeoramiento en varias de las dimensiones estudiadas, comparado con su situación antes de padecer la enfermedad.

También, nuestros hallazgos son compatibles con los identificados por una investigación de Brasil por cuanto informan que las principales repercusiones sobre la CVRS se encuentran en el dominio rol físico, evaluado por el instrumento SF-36 30.

Otro estudio, llevado a cabo en Bélgica, en el que se utilizó el instrumento SF-36 para evaluar CVRS, evidenció deterioro en los resultados específicos de calidad de vida para los dominios asociados a la salud física de los sobrevivientes de la COVID-19, principalmente debido a los requerimientos de ventilación mecánica asociados a la estancia en UCI 31.

Con respecto a la funcionalidad, una revisión sistemática demostró que aproximadamente el 90% de los pacientes hospitalizados por COVID-19 experimentan secuelas, sobre todo a nivel respiratorio y funcional 32. Hasta el momento, la evidencia demuestra que la mayoría de los pacientes sobrevivientes de COVID-19 tienen síntomas persistentes al menos hasta seis meses después del alta hospitalaria 8,12,18.

Este estudio evidenció la persistencia de síntomas limitantes y no limitantes, principalmente fatiga, disnea, dolor, caída de cabello, irritabilidad, insomnio y tos tras el egreso. Este hallazgo también ha sido descrito por otros autores 3-5,33-37 que resaltan la fatiga, la disnea, la tos y la debilidad muscular como síntomas persistentes presentes hasta siete meses tras el alta hospitalaria 38.

Otra cuestión de importancia es que muchas de las personas afectadas por la enfermedad se enfrentan a episodios de ansiedad y síntomas depresivos derivados del miedo a fallecer, la incertidumbre, el aislamiento social y el deterioro de la productividad 2,39-40. Estos resultados también fueron evidenciados en nuestro estudio, donde gran parte de los participantes aún tenían sentimientos de ansiedad y síntomas relacionados con la depresión. Sin embargo, cabe aclarar que existen estudios que encontraron puntuaciones bajas, es decir, una mayor alteración en los ítems relacionados con estado emocional y social 2,4, aunque nuestros hallazgos no reportan efectos negativos en estas dimensiones.

Diferentes investigaciones que indagaron sobre el estado funcional en la etapa post-COVID-19 haciendo uso del instrumento índice de Barthel, mencionan la importante dependencia física que se deriva de la COVID-19 incluso hasta los tres meses posteriores al alta hospitalaria 10,11,41. Nuestros resultados se suman a estos hallazgos por cuanto en nuestro estudio solo el 9% de los participantes negaron algún tipo de limitación funcional.

Ahora bien, el uso de la escala PCFS también ha demostrado la afectación sobre el estado funcional en las personas que han tenido COVID-19 y han requerido cuidado en una institución de salud 34,36,42. Este estudio identificó que el 91% de los participantes presentaba algún grado de limitación evaluada por la escala PCFS (mínima, ligera, moderada o severa), hallazgo que se asemeja al de un estudio francés 4, el cual determinó que la mayoría de los pacientes que requirieron hospitalización por COVID-19 presentaron síntomas que conllevaron una limitación física para la ejecución de actividades varios meses después del alta.

Asimismo, nuestros resultados se asemejan a los de otro estudio realizado en Dinamarca con una muestra similar, en el cual encontraron un deterioro leve a moderado en el estado funcional percibido por los participantes tras la infección por COVID-19 34.

En la evidencia también se encuentran otras investigaciones que han indagado tanto sobre la CVRS como sobre el estado funcional 10-17. Por ejemplo, una investigación llevada a cabo en España 10 demuestra el deterioro sobre la función física, pero a diferencia de nuestros hallazgos, en lo que respecta a la CVRS, indica una mayor afectación derivada en la etapa post-COVID-19. Esto pudo deberse a las características de la muestra, ya que en nuestro estudio se incluyeron pacientes que requirieron manejo hospitalario en UCI, UCIN y en hospitalización general, con un promedio de días de estancia hospitalaria de 28,46, a diferencia del estudio en mención, para el cual todos los participantes estuvieron en UCI con un promedio de días de estancia hospitalaria de 53.

Otro estudio desarrollado en Bélgica evaluó tanto CVRS como estado funcional con los instrumentos EQ-5D-3L e índice de Barthel, respectivamente. Sus hallazgos sugieren también una reducción significativa de la CVRS en los participantes y un importante porcentaje de participantes con dependencia al año de la enfermedad grave 11.

De manera similar, en otra investigación que evaluó tanto calidad de vida como estado funcional, se encontraron deterioros en ambas percepciones por parte de los sobrevivientes de COVID-19, incluso aquellos que padecieron de una forma leve de la enfermedad 12.

Nuestro estudio pudo determinar tanto la presencia de síntomas que condicionan la funcionalidad como la estrecha relación que estos tienen con la percepción de la calidad de vida de quienes fueron diagnosticados y tratados por COVID-19 en instituciones de salud, ya que a mayor número de síntomas limitantes peor fue la percepción de calidad de vida 33.

Al igual que en nuestro estudio, una investigación desarrollada en España 42 para evaluar las características psicométricas de la Escala Española de Estado Funcional Post-COVID-19 (PCFS), mostró una alta correlación inversa con un instrumento que evalúa la CVRS: el EQ-5D-5L, evidenciando un deterioro de la calidad de vida a mayor nivel de limitación funcional.

Cabe aclarar que, en general, la mayoría de los estudios que indagan sobre CVRS y estado funcional han utilizado instrumentos diferentes a los utilizados en nuestra investigación 10,12-14,34,41-42. No obstante, puede afirmarse que los hallazgos son congruentes con lo reportado en la literatura hasta ahora. Las condiciones físicas, psicológicas, sociales y económicas derivadas de la enfermedad parecen traer consigo un alto potencial de impacto negativo sobre la calidad de vida y el estado funcional de las personas afectadas, lo que demuestra que la COVID-19 tiene consecuencias significativas más allá de la etapa aguda y crítica de la enfermedad.

Los resultados mostraron una manifestación más grave de la COVID-19 en hombres, quienes requirieron cuidado en la UCI con más frecuencia que las mujeres. De igual forma, el hábito de fumar demostró ser un factor predisponente de complicación en comparación con las personas no fumadoras.

Se identificó que la COVID-19 tiene un impacto importante en la percepción de la calidad de vida, incluso más allá de seis meses tras el alta hospitalaria. Un número importante de participantes refirieron sentirse cansados y exhaustos con frecuencia, además de expresar problemas en el desarrollo de actividades de la vida diaria, incluyendo el trabajo, y evaluaron con frecuencia la salud propia como regular, con posibilidad de que esta empeore. Con respecto a la salud mental, los pacientes en la etapa post COVID-19 refirieron tener sentimientos de angustia y síntomas depresivos a menudo. Adicionalmente, se identificó la presencia de dolor que podía llegar a ser limitante en la ejecución de actividades cotidianas o laborales. En contraste, no se evidenció mayor afectación en la capacidad de los participantes para participar de actividades sociales.

Se identificó en los participantes una percepción de salud general en el momento de la encuesta más o menos igual que la de hace un año. Sin embargo, el 21% de ellos manifestaron que era algo o mucho peor su salud percibida ahora.

Por otro lado, es evidente el efecto de la COVID-19 sobre la funcionalidad de quienes lo han vivido, ya que solo el 9% de los participantes refirieron no tener ningún tipo de limitación funcional, y el porcentaje restante presentó limitaciones que varían de leves a severas. Síntomas como fatiga, disnea y dolor en miembros inferiores fueron los síntomas limitantes hallados con mayor frecuencia, mientras que la tos, la caída de cabello, la irritabilidad y el insomnio fueron síntomas no limitantes a menudo expresados.

Finalmente, un análisis de correlación permitió determinar que la calidad de vida percibida por los participantes estuvo mediada por la funcionalidad física, siendo peor la percepción de la CVRS a medida que se aumenta el grado de limitación funcional ♠
